# Impact of Papillary Morphology and Diverticular Type on Needle-Knife Papillotomy in Patients with Periampullary Diverticulum with Difficult Biliary Cannulation

**DOI:** 10.3390/jcm14228208

**Published:** 2025-11-19

**Authors:** Kuan-Ting Liu, Sheng-Fu Wang, Chi-Huan Wu, Mu-Hsien Lee, Yung-Kuan Tsou, Cheng-Hui Lin, Kai-Feng Sung, Nai-Jen Liu

**Affiliations:** 1 Department of Gastroenterology and Hepatology, Chang Gung Memorial Hospital, Taoyuan 333, Taiwan; a3506377@cgmh.org.tw (K.-T.L.); shanelily@msn.com (S.-F.W.); b9002076@cloud.cgmh.org.tw (C.-H.W.); r5266@cloud.cgmh.org.tw (M.-H.L.); linchehui@cloud.cgmh.org.tw (C.-H.L.); h12153@cloud.cgmh.org.tw (K.-F.S.); milk1372@cloud.cgmh.org.tw (N.-J.L.); 2 Department of Medicine, College of Medicine, Chang Gung University, Taoyuan 333, Taiwan

**Keywords:** periampullary diverticulum, needle-knife papillotomy, major duodenal papilla morphology, endoscopic retrograde cholangiopancreatography

## Abstract

**Background/Objectives:** While previous studies have explored the relationship between periampullary diverticulum (PAD) and conventional endoscopic retrograde cholangiopancreatography (ERCP) success, data on advanced cannulation techniques like needle-knife papillotomy (NKP) remain limited. This study aimed to assess NKP outcomes in PAD patients with difficult biliary cannulation. **Methods:** A retrospective study was conducted on 122 PAD patients who underwent NKP in a single center. Patient characteristics, ERCP indications, common bile duct diameter, PAD type, diverticular size, major duodenal papilla (MDP) morphology, and post-ERCP adverse events were assessed. We also analyzed factors associated with the outcomes of NKP in patients with PAD. **Results:** Of the 122 patients, NKP was successful in 82 (67.2%) and failed in 40 (32.8%), with diverticular diameter being significantly larger in the failure group. By PAD type, the diverticular median diameters were 1.2 cm (type I), 0.9 cm (type II), and 0.5 cm (type III) (*p* < 0.001), with NKP success rates of 50%, 66.3%, and 75%, respectively (*p* = 0.391). By MDP morphology, the success rates were 73.7% (type I), 38.2% (type II), 92.9% (type III), and 82.4% (type IV) (*p* = 0.059). The overall adverse event rate was 16.4%, with pancreatitis (6.6%), bleeding (5.7%), and cholangitis (4.1%) showing no significant differences between the success and failure groups. Multivariate analysis identified MDP morphology (type II vs. I, OR: 0.256, *p* = 0.011) and active bleeding during NKP (OR: 0.117, *p* < 0.001) as independent predictors of failure. **Conclusions:** MDP morphology and intraprocedural bleeding are significant independent predictors of NKP failure in PAD patients with difficult biliary cannulation, whereas PAD type has no significant impact on NKP outcomes.

## 1. Introduction

The successful performance of endoscopic retrograde cholangiopancreatography (ERCP) in treating biliary diseases relies significantly on the crucial step of selective biliary cannulation (SBC). Nevertheless, even experienced endoscopists encounter a failure rate of 5–15% when employing conventional biliary cannulation methods for SBC [1]. One reported contributing factor to this failure is the presence of a periampullary diverticulum (PAD) [2], which can influence the outcome of ERCP by potentially altering the location and orientation of the ampulla. Notably, the literature reports varying rates of successful SBC in the presence of PAD, ranging from 64.5% to 89.4% [3,4,5]. The type of PAD may account for the wide variation in SBC success rates [5,6], with three classifications existing in the literature based on the major papilla’s location relative to the diverticulum [6,7,8]. Among these classifications, the Boix classification, encompassing three PAD types, is likely the most commonly employed [8].

For patients encountering difficulty with conventional cannulation methods due to PAD, guidelines propose precut sphincterotomy as an alternative cannulation technique [9]. However, needle-knife precut sphincterotomy (NKPS), which includes needle-knife papillotomy (NKP) and needle-knife fistulotomy (NFK), does not consistently achieve SBC [10,11]. Our previous study indicated a significant association between PAD and NKP failure in univariate analysis, although not in multivariate analysis [12]. Given the variability of PAD among patients, the type of PAD may influence the success of NKPS in PAD patients. Additionally, as the major duodenal papilla (MDP) serves as the gateway to the common bile duct (CBD), the MDP morphology may also impact the SBC rate in PAD patients undergoing NKPS [13,14,15]. Despite this importance, there are currently no reports on the influence of PAD type and MDP morphology in PAD patients undergoing NKPS due to difficult biliary cannulation. Therefore, this study aims to analyze factors associated with the outcomes of NKP in patients with PAD, with a specific focus on PAD type and MDP morphology.

## 2. Patients and Methods

**Figure 1:**
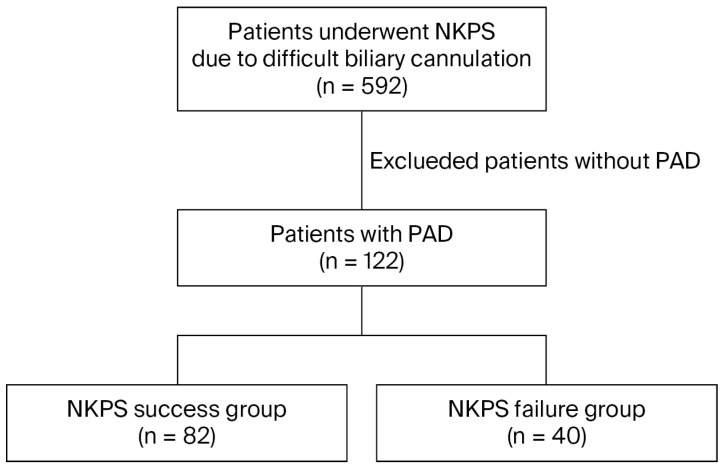
The study flowchart. Abbreviations: NKPS: needle-knife precut sphincterotomy; PAD: periampullary diverticulum.

Figure 1 shows the study’s flowchart. A total of 592 patients who underwent NKP due to difficult bile duct cannulation (described below) during ERCP between January 2004 and December 2020 were retrospectively selected from the database of our institution’s Therapeutic Endoscopic Center. Among them, 122 patients (20.6%) with PAD (from a total of 2236 PAD patients who underwent ERCP during the study period) were included in this study. The patients were divided into two groups: the NKP success group, in which SBC was successfully achieved after NKP, and the NKP failure group, where SBC could not be achieved despite NKP. Comprehensive data, encompassing patient characteristics (age, gender), ERCP indications (choledocholithiasis, benign or malignant stricture, and bile leakage), CBD diameter, bleeding during NKP, MDP morphology, PAD classification, diverticular size, and post-ERCP adverse events (pancreatitis, cholangitis, bleeding, and perforation), were extracted from medical and imaging records.

### 2.1. ERCP and NKP Procedures

All procedures were performed by five endoscopists (A–E). Over the past 10 years, the average annual ERCP case numbers for endoscopists A, B, C, D, and E were approximately 300, 200, 200, 200, and 130, respectively. Only endoscopist A, who had been performing ERCP since 1995, had NKP experience before the study. Since endoscopists B, C, D, and E learned the ERCP/NKP technique from endoscopist A, all NKPS procedures in this study involved NKP.

The details of ERCP and NKP procedures were described in our previous study [12]. In brief, the initial selective cannulation of the CBD was performed using either a cannula or a pull-type sphincterotome, based on the endoscopist’s preference. Because insurance coverage limited the number of guidewires to one, only some endoscopists occasionally resorted to the double-guidewire method after the initial two approaches failed to achieve SBC (less than 5% per endoscopist). As a result, only 5 patients underwent the double-guidewire method before NKP. Difficult biliary cannulation was defined when all the aforementioned methods failed to achieve SBC. There were no strict rules regarding the timing and number of biliary cannulations, nor the number of pancreatic duct cannulations; these were determined by the endoscopist. However, from 2015 onwards, the endoscopists often adopted an early precut strategy [12].

NKP was performed for patients with difficult biliary cannulation during the same session as ERCP using a needle-knife sphincterotome (Rx Needle-Knife XL; Boston Scientific Corporation, Marlborough, MA, USA). After puncturing the papilla above the orifice, an incision was made upward from the papillary orifice along the bile duct axis. The incision was extended until the CBD was exposed (but not beyond the uppermost part of the papilla), followed by a small incision in the biliary sphincter muscle. The CBD was then cannulated directly with the closed needle-knife or with a wire-guided cannula/sphincterotome. A successful NKP procedure was defined by the deep placement of a catheter or sphincterotome into the CBD with the acquisition of a satisfactory cholangiogram. After achieving deep cannulation, a pull-type sphincterotomy was used to extend the incision towards the duodenal wall superiorly. Whether to place a pancreatic duct stent before NKP depended on whether the pancreatic duct had been cannulated and the judgment of the endoscopist.

The definition of procedure-related adverse events was based on the ASGE lexicon for endoscopic adverse events [16]. Because rectal nonsteroidal anti-inflammatory drugs (NSAIDs) were not available in our endoscopy suite during the study period, they were not used to prevent post-ERCP pancreatitis [17].

### 2.2. Classification of Periampullary Diverticulum

**Figure 2:**
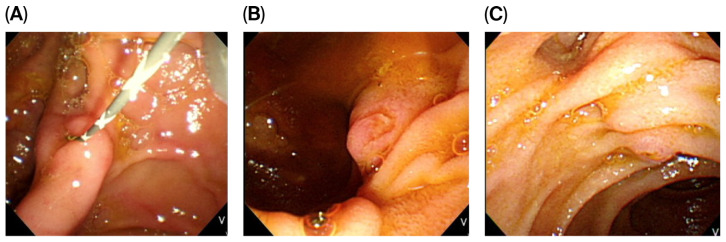
Based on Boix’s classification, type I papillae were within the diverticulum (**A**), type II were at the margin of the diverticulum (**B**), type III were near the diverticulum (**C**).

PAD was divided into three types according to the Boix classification [8]. Type I papillae were within the diverticulum (Figure 2A); type II were at the margin of the diverticulum (Figure 2B); type III were near the diverticulum (Figure 2C). We measured the PAD diameter from endoscopic images using a cannula or sphincterotome as a comparison caliper.

### 2.3. Morphology of the Major Duodenum Papilla

**Figure 3:**
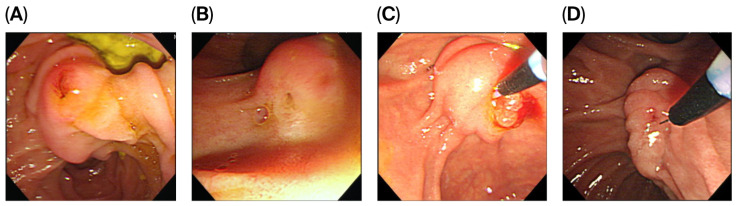
Based on the classification proposed by the Scandinavian group, the morphology of MDP was divided into four types: type I, regular papilla (**A**); type II, small papilla (**B**); type III, protruding or pendulous papilla (**C**); type IV, creased or ridged papilla (**D**).

According to the classification proposed by the Scandinavian group, MDP morphology was divided into four types: type I, regular papilla (Figure 3A); type II, small papilla (Figure 3B); type III, protruding or pendulous papilla (Figure 3C); type IV, creased or ridged papilla (Figure 3D) [14].

### 2.4. Statistical Analysis

In both the text and tables, continuous variables are presented as medians with ranges, while categorical variables are expressed as numbers (percentages). To compare the NKP success and failure groups, the Mann–Whitney U test was employed for continuous variables, and Chi-square or Fisher’s exact tests were used for categorical variables. Logistic regression analysis was conducted to identify factors associated with NKP success or failure, and a two-tailed *p*-value of <0.05 was considered statistically significant. All statistical analyses were executed using Statistical Product and Service Solutions (SPSS, version 26, IBM, Armonk, NY, USA).

## 3. Results

A total of 122 patients participated in the study, with 82 (67.2%) classified in the NKP success group and 40 (32.8%) in the NKP failure group.

### 3.1. Result Comparisons Between the NKP Success and Failure Groups

Table 1 outlines comprehensive baseline characteristics of all included patients. The median age was 75 years, with 49.2% being male, and no significant differences in age or gender were noted between the two groups. Choledocholithiasis was the predominant ERCP indication in 86.1% of cases, with no significant intergroup variations for each indication. The median CBD diameter was 1.1 cm and did not show a significant difference between the success and failure groups. Diverticula were categorized as type I (8/122 or 6.6%), type II (86/122 or 70.5%), and type III (28/122 23%), with no statistically significant differences between the two groups. The median diverticulum diameter was 0.8 cm; however, it was significantly larger in the NKP failure group (0.8 cm vs. 1 cm, *p* = 0.001). MDP types I, II, III, and IV had incidence rates of 46.7%, 27.9%, 11.5%, and 13.9%, respectively. Among the four types, the proportion of type II MDP was significantly lower in the NKP success group (15.9% vs. 52.5%, *p* < 0.001), while that of type III was significantly higher (15.9% vs. 2.5%, *p* = 0.03). Immediate bleeding during primary ERCP occurred in 22.9% of patients and more frequently in the NKP failure group (12.2% vs. 45%, *p* < 0.001). Thirty-two patients (26.2%) underwent pancreatic stenting. Pancreatic stent placement was at the discretion of each endoscopist, and there was no significant difference in stenting frequency between the NKP success and failure groups (28% vs. 22.5%, *p* = 0.662). Among the patients with surgically altered anatomy (five in total), four were in the NKP success group and one was in the NKP failure group (*p* = 0.736). The overall adverse event rate was 16.4%, including pancreatitis (6.6%), delayed bleeding (5.7%), and cholangitis (4.1%). The incidence of pancreatitis was 4.9% and 10% in the NKP success and failure groups (*p* = 0.283); delayed bleeding’s incidence was 6.1% and 5% in the NKP success and failure groups (*p* = 0.807); and cholangitis occurred in 4.9% and 2.5% of the NKP success and failure groups (*p* = 0.534), respectively. Notably, there were no instances of perforation in either group.

Among the 40 NKP failure group patients, 11 underwent a second ERCP, of which 9 were successful. Two patients who failed underwent a third ERCP that still failed; both patients underwent percutaneous transhepatic biliary drainage (PTCD). After initial ERCP failure, 15 and 6 patients underwent PTCD and surgery, respectively, while the remaining 8 patients refused further treatment and received only conservative treatment with antibiotics.

### 3.2. Results Based on Diverticular Type

Table 2 summarizes patient characteristics and outcomes based on diverticular type. Type I PAD patients are predominantly male (87.5%), while type II and III diverticula are evenly distributed between males and females (*p* = 0.074). Regarding the indications for ERCP, the main indication for type I PAD was malignant biliary stricture (75%), whereas the main indication for types II and III PAD was choledocholithiasis (88.4% and 78.6%, respectively) (*p* = 0.076). There was no significant difference in median CBD diameter between each PAD type, though there were significant differences in diverticula size between PAD types, with sizes of 1.2 cm, 0.9 cm, and 0.5 cm in type I, II, and III PAD, respectively (*p* < 0.001). Type III PAD had the highest NKP success rate (75%), followed by type II PAD (66.3%), and type I PAD had the lowest SBC success rate (50%); however, these differences did not reach statistical significance (*p* = 0.391). The occurrence rates of adverse events, such as pancreatitis (0%, 8.1%, and 3.6% for PAD types I, II, and III, respectively; *p* = 0.164), delayed bleeding (12.5%, 8.1%, and 3.6% for PAD types I, II, and III, respectively; *p* = 0.225), and cholangitis (0%, 1.2%, and 14.3% for PAD types I, II, and III, respectively; *p* = 0.365), showed no significant differences by PAD type.

### 3.3. Results Based on Papillary Morphology

Table 3 presents outcomes with different major papilla morphologies. Immediate bleeding’s incidence during NKP was 19.3%, 26.5%, 21.4%, and 29.4% for MDP types I, II, III, and IV, respectively (*p* = 0.29), and the overall NKP success rate was 73.7%, 38.2%, 92.9%, and 82.4% for patients with MDP types I, II, III, and IV, respectively (*p* = 0.059). The occurrence rates of adverse events, such as pancreatitis (7%, 2.9%, 0%, and 17.6% for MDP types I, II, III, and IV, respectively; *p* = 0.162), delayed bleeding (5.3%, 2.9%, 14.3%, and 5.9% for MDP types I, II, III, and IV, respectively; *p* = 0.492), and cholangitis (3.5%, 5.9%, 7.1%, and 0% for MDP types I, II, III, and IV, respectively; *p* = 0.71), showed no significant differences by MDP type.

### 3.4. Factors Associated with NKP Outcomes

Univariate and multivariate analyses were performed to identify factors associated with NKP outcomes, as shown in Table 4. Univariate analysis showed that MDP morphology (type II vs. type I, odds ratio [OR]: 0.221; 95% confidence interval [CI]: 0.089–0.549]; *p* = 0.001), diverticulum size (OR: 0.213; 95% CI: 0.082–0.555; *p* = 0.002), and bleeding during NKP (OR: 0.170; 95% CI: 0.068–0.421; *p* < 0.001) were significantly associated with NKP outcome. Multivariate analysis showed that MDP morphology (type II vs. type I, OR: 0.256; 95% CI: 0.089–0.734; *p* = 0.011) and bleeding during NKP (OR: 0.117; 95% CI: 0.039–0.351; *p* < 0.001) were independent factors associated with NKP outcome.

## 4. Discussion

PAD is a factor that has been inconsistently linked to the success rate of SBC in patients undergoing ERCP [3,4,5]. As precut sphincterotomy in difficult cases increases the overall SBC success rate by 25% points, this study adds valuable insights by examining the influence of PAD classification and MDP morphology on NKP outcomes [9].

There is little data regarding precut sphincterotomy in patients with PAD [18,19]. Arabpour et al. reported that for patients with PAD and difficult biliary cannulation, NKF, NKP, or transpancreatic biliary sphincterotomy (TPS) can all be used as rescue methods [20]. However, they did not evaluate the success rate of each precut method. Park et al. reported that among 33 patients with PAD who underwent NKF, type II PAD was the most common and type I was the least common, which is similar to our findings [19]. However, due to the small number of cases, they did not analyze the success rate of each type [19]. Our results show that type III PAD had the highest (75%) and type I had the lowest success rate (50%). This may be because the MDP is located outside the diverticulum in type III PAD and is therefore minimally affected by the PAD, allowing the endoscopist to perform NKP as normal, with the highest success rate. In comparison, PAD types I and II have more papillae that are difficult to detect or access, or bile duct directions that are more unpredictable [21]. Fernandes et al. compared NKF for flat, intradiverticular (corresponding to type I PAD, *n* = 14), and diverticular marginal papillae (corresponding to type II PAD, *n* = 14), and reported success rates of 93.9%, 64.3%, and 71.4% (*p* = 0.005), respectively [18]. They concluded that NKF is feasible in diverticular papillae but the initial ERCP success rate is relatively low, which is consistent with our results.

The clinical influences of PAD size have rarely been studied. Kim et al. reported that CBD diameter was significantly related to PAD size and that patients with type I PAD had significantly larger CBD diameters than type III patients [22]. That is, type I PAD presented as larger while type III PAD tended to be smaller [22]. These findings were consistent with our results showing that type I PADs were significantly larger in size, followed by type II, and type III were significantly smaller. Furthermore, we found that the NKP success group had a significantly smaller PAD size. However, the precise determination of PAD size is not straightforward. In our study, PAD diameter was measured through endoscopic imaging, whereas other studies used CT or MRCP, resulting in the median PAD diameter in our study being smaller than in other reports [22].

Several studies have explored the influence of MDP morphology on the technical success of ERCP [14,15,23]. Since its introduction in 2017, the Scandinavian classification of MDP morphology has gradually gained acceptance due to its remarkable intra- and inter-observer agreement [9,14,23,24]. Based on this classification, Haraldsson et al. found that cannulating type II and III MDPs is more difficult [23]. However, the Scandinavian classification lacks some important papillary subtypes, such as those associated with PAD. Nevertheless, the inclusion of type IV to account for PAD, as reported by Mohamed et al., yielded similar findings, indicating increased difficulty in cannulating type II and III MDPs compared to type I [15]. Additionally, they observed a higher frequency of NKP in types II, III, and IV MDP compared to type I (*p* < 0.001). Our study further explored the correlation between MDP morphology and NKP outcomes in patients with PAD: type II MDP had the lowest NKP success rate (38.2%), whereas type III MDP had the highest (92.9%). Because most endoscopists consistently perform the same type of precut sphincterotomy over time, different precut techniques based on MDP morphology may be considered to improve success rates in patients with PAD, especially type II MDP [9,25,26].

Several studies have suggested a potential link between MDP type and adverse events during ERCP [15]. However, in a study by Lyu et al. comparing the safety of NKP among various MDP types in patients with difficult biliary cannulation (8.3% of patients had PAD), no significant difference was found in the incidence of adverse events among the four MDP types [27]. Our findings were in line with this, albeit limited to patients with PAD. Additionally, our study revealed no significant differences in adverse event rates among the three types of PAD. Notably, perforation did not occur in our study, potentially due to its small sample size. Therefore, larger-scale studies may be necessary in the future.

This study is subject to various limitations. First, it was conducted retrospectively and involved a sample in which procedures were carried out by five endoscopists. Therefore, selection and operator bias may exist, limiting the general applicability of the results. Second, although the incidence of adverse events in this study is consistent with previous studies, the limited sample size could potentially impact the results. Due to the unavailability of rectal NSAIDs, they were not used in this study, which may have influenced the incidence of post-ERCP pancreatitis. However, as noted in the ASGE guidelines, the routine prophylactic use of rectal NSAIDs in clinical practice remains suboptimal [17]. In addition, because pancreatic stents can reduce the risk of pancreatitis after ERCP and may improve NKP outcomes [28], the utilization rate of pancreatic stents was 26.2%, which may have affected the results of this study. Third, because only NKP was performed in this study, our results may not be directly applicable to other precut methods such as NKF or TPS. Fourth, the number of type I diverticulum cases was relatively small. One reason for this may be that in patients whose MDP could not be reached using conventional ERCP cannulation techniques, we typically employed endoclip-assisted biliary cannulation or the two-devices-in-one-channel method, both of which usually resulted in successful biliary cannulation. Therefore, large-scale, multicenter, prospective studies are needed in the future to validate our findings.

In conclusion, type II MDP morphology and significant bleeding during NKP were significantly associated with NKP failure in PAD patients with difficult bile duct cannulation, whereas type III MDP morphology showed a trend toward increased NKP success. These findings may have important implications for ERCP practitioners, particularly in patients with type II MDP morphology, in whom techniques other than NKP may be considered.

## Figures and Tables

**Table 1:** Characteristics of the patients in the NKPS success and failure groups.

	Overall (*n* = 122)	NKPS Success Group (*n* = 82)	NKPS Failure Group (*n* = 40)	*p*-Value
Age, years (range)	75 (35–94)	75 (35–94)	76 (51–92)	0.654
Male, *n*	60 (49.2%)	42 (51.2%)	18 (45%)	0.519
Indication of ERCP, *n*				
Choledocholithiasis	105 (86.1%)	70 (85.3%)	35 (87.5%)	0.585
Malignant stricture	11 (9%)	7 (8.5%)	4 (10.0%)	0.235
Benign stricture	3 (2.5%)	2 (2.4%)	1 (2.5%)	0158
Bile leakage	3 (2.5%)	3 (8.5%)	0 (0.0%)	0.244
CBD diameter, cm	1.1 (0.3–3.0)	1.1 (0.3–2.7)	1.0 (0.4–3.0)	0.336
Type of diverticulum, *n*				
Type I	8 (6.6%)	4 (4.9%)	4 (10.0%)	0.179
Type II	86 (70.5%)	57 (69.5%)	29 (72.5%)	0.078
Type III	28 (23.0%)	21 (25.6%)	7 (17.5%)	0.123
Diverticulum diameter, cm	0.8 (0.2–2)	0.8 (0.2–2)	1.0 (0.3–1.9)	0.001
Morphology of papilla, *n*				
Type I	57 (46.7%)	42 (51.2%)	15 (37.5%)	0.154
Type II	34 (27.9%)	13 (15.9%)	21 (52.5%)	<0.001
Type III	14 (11.5%)	13 (15.9%)	1 (2.5%)	0.030
Type IV	17 (13.9%)	14 (17.1%)	3 (7.5%)	0.158
Bleeding during NKPS, *n*	28 (22.9%)	10 (12.2%)	18 (45.0%)	<0.001
Pancreatic stent	32 (26.2%)	23 (28.0%)	9 (22.5%)	0.662
Surgically altered anatomy, *n*	5	4	1	0.736
B-II anastomosis	4 (3.3%)	3 (3.7%)	1 (2.5%)	0.689
Roux-en-Y anastomosis	1 (0.8%)	1 (1.2%)	0	0.998
Adverse events of ERCP, *n*				
Overall	20 (16.4%)	13 (15.9%)	7 (17.5%)	0.485
Pancreatitis	8 (6.6%)	4 (4.9%)	4 (10.0%)	0.283
Delayed bleeding	7 (5.7%)	5 (6.1%)	2 (5.0%)	0.807
Cholangitis	5 (4.1%)	4 (4.9%)	1 (2.5%)	0.534
Perforation	0	0	0	0.999

Abbreviations: ERCP: endoscopic retrograde cholangiopancreatography; CBD: common bile duct; NKPS: needle-knife precut sphincterotomy; B-II: Billroth-II.

**Table 2:** Patient characteristics and outcomes based on diverticular type.

	Type I (*n* = 8)	Type II (*n* = 86)	Type III (*n* = 28)	*p*-Value
Age, years	69.5 (69–74.8)	77 (66–84)	72 (62–79)	0.188
Male, *n* (%)	7 (87.5%)	39 (45.3%)	14 (50%)	0.074
Indication of ERCP, *n*				
Choledocholithiasis	1 (12.5%)	76 (88.4%)	22 (78.6%)	0.088
Malignant stricture	6 (75.0%)	7 (8.1%)	3 (10.7%)	0.136
Benign stricture	1 (12.5%)	1 (1.2%)	2 (7.1%)	0.255
Bile leakage	0	2 (2.4%)	1 (3.6%)	0.263
CBD diameter, cm	1.4 (0.5–2.8)	1.1 (0.3–3.0)	1.0 (0.6–8.0)	0.859
Diverticulum diameter, cm	1.2 (0.5–1.9)	0.9 (0.3–2.0)	0.5 (0.2–1.9)	<0.001
NKPS success, *n*	4 (50%)	57 (66.3%)	21 (75%)	0.3910
Adverse events, *n*				
Overall	1 (12.5%)	15 (17.4%)	6 (21.5%)	0.385
Pancreatitis	0	7 (8.1%)	1 (3.6%)	0.164
Delayed bleeding	1 (12.5%)	7 (8.1%)	1 (3.6%)	0.225
Cholangitis	0	1 (1.2%)	4 (14.3%)	0.365

Abbreviations: ERCP: endoscopic retrograde cholangiopancreatography; CBD: common bile duct; NKPS: needle-knife precut sphincterotomy.

**Table 3:** Outcomes based on major duodenal papilla morphology.

	Type I (*n* = 57)	Type II (*n* = 34)	Type III (*n* = 14)	Type IV (*n* = 17)	*p*-Value
Bleeding during NKPS	11 (19.3%)	9 (26.5%)	3 (21.4%)	5 (29.4%)	0.290
NKPS success, *n*	42 (73.7%)	13 (38.2%)	13 (92.9%)	14 (82.4%)	0.059
Adverse events, *n*					
Overall	9 (15.8%)	4 (11.7%)	3 (21.4%)	4 (23.5%)	0.884
Pancreatitis	4 (7.0%)	1 (2.9%)	0	3 (17.6%)	0.162
Delayed bleeding	3 (5.3%)	1 (2.9%)	2 (14.3%)	1 (5.9%)	0.492
Cholangitis	2 (3.5%)	2 (5.9%)	1 (7.1%)	0	0.710

Abbreviations: NKPS: needle-knife precut sphincterotomy.

**Table 4:** Univariate and multivariate analyses of the factors associated with needle-knife precut sphincterotomy success or failure.

Variables		Univariate Analysis		Multivariate Analysis	
		OR (95% CI)	*p*-Value	OR (95% CI)	*p*-Value
Age	>70 y	1.071 (0.486–2.361)	0.864		
	≤70 y	Referent			
Gender	Male	1.283 (0.601–2.740)	0.519		
	Female	Referent			
Choledocholithiasis	Yes	0.500 (0.054–4.626)	0.570		
	No	Referent			
Malignant biliary stricture	Yes	0.840 (0.231–3.055)	0.791		
	No	Referent			
CBD diameter (cm)	>6 mm	1.029 (0.327–3.239)	0.962		
	≤6 mm	Referent			
Morphology of papilla	Type II	0.221 (0.089–0.549)	0.001	0.331 (0.123–0.887)	0.028
	Type III	4.643 (0.559–38.590)	0.155	9.122 (0.886–93.924)	0.063
	Type IV	1.667 (0.420–6.620)	0.468	2.66 (0.572–12.373)	0.212
	Type I	Referent		Referent	
Type of diverticulum	Type I	0.333 (0.065–1.699)	0.402		
	Type II	0.655 (0.250–1.720)	0.502		
	Type III	Referent			
Diverticulum diameter	Every 1 cm increase	0.213 (0.082–0.555)	0.002	0.408 (0.252–1.121)	0.202
Surgically altered anatomy	Yes	1.481	0.737		
	No	Referent		Referent	
Bleeding during NKPS	Yes	0.170 (0.068–0.421)	<0.001	0.117 (0.039–0.351)	<0.001
	No	Referent		Referent	
Endoscopist	B + C + D + E	0.898	0.688		
	A	Referent			
Pancreatic stent	Yes	1.343 (0.554–3.253)	0.514		
	no	Referent			

Abbreviations: CBD: common bile duct; NKPS: needle-knife precut sphincterotomy; OR: odds ratio; CI: confidence interval.

## Data Availability

Deidentified individual participant data are available and will be provided on reasonable request to the corresponding author.

## Abbreviations

The following abbreviations are used in this manuscript:

PAD	periampullary diverticulum
ERCP	endoscopic retrograde cholangiopancreatography
NKP	needle-knife papillotomy
MDP	major duodenal papilla
SBC	selective biliary cannulation
NKPS	needle-knife precut sphincterotomy
CBD	common bile duct
NSAIDs	nonsteroidal anti-inflammatory drugs
